# Supplementary material for: Dual electrical stimulation at spinal-muscular interface reconstructs spinal sensorimotor circuits after spinal cord injury
Source: Nat Commun. 2024 Jan 19;15:619. doi: 10.1038/s41467-024-44898-9 (PMC10799086; doi:10.1038/s41467-024-44898-9)
Supplement: Supplementary file 1 — Supplementary Information [file 41467_2024_44898_MOESM1_ESM.pdf]

Supplementary Information for "Dual electrical stimulation at spinal-muscular interface reconstructs spinal sensorimotor circuits after spinal cord injury"

Kai Zhou[1,2+], Wei Wei[1+], Dan Yang[1,3+], Hui Zhang[1], Wei Yang[1], Yunpeng Zhang[4], Yingnan Nie[5], Mingming Hao[6,7], Pengcheng Wang[8], Hang Ruan[8], Ting Zhang[6], Shouyan Wang[5], Yaobo Liu[1,2*]

Affiliations:

1. Jiangsu Key Laboratory of Neuropsychiatric Diseases and Institute of Neuroscience, Soochow University; Clinical Research Center of Neurological Disease, The Second Affiliated Hospital of Soochow University, Suzhou 215123, China.
2. Co-innovation Center of Neuroregeneration, Nantong University, Nantong 226001, China.
3. Department of Anatomy, School of Basic Medical Science, Guizhou Medical University, Guiyang 550025, China.
4. Suzhou Institute of Biomedical Engineering and Technology, Chinese Academy of Sciences, Suzhou, Jiangsu 215163, China.
5. Institute of Science and Technology for Brain-Inspired Intelligence, Fudan University, Shanghai 200433, China.
6. *i*-Lab, Key Laboratory of Multifunctional Nanomaterials and Smart Systems, Suzhou Institute of Nano-tech and Nano-bionics, Chinese Academy of Sciences, Suzhou, Jiangsu 215123, China.
7. Ningbo Medical Centre Lihuili Hospital, Ningbo, Zhejiang, 315048, P. R. China.
8. Institutes of Biology and Medical Sciences, Soochow University, Suzhou, Jiangsu 215123, China.

[+] These authors contributed equally to this work.

* Corresponding author. Email: liuyaobo@suda.edu.cn

Supplementary table 1. Key resources in this study

Reagent type (species) or resource	Designation	Source or reference	Identifiers	Additional information
strain, strain background (Mus Musculus)	ChAT-iCre	The Jackson Laboratory	JAX#006410	
strain, strain background (Mus Musculus)	Lbx1-iCre	GemPharmatech	This paper	Genetically altered mice were used to label interneurons
strain, strain background (Mus Musculus)	C57BL/6J	SLAC Laboratory Animal Company		
antibody	Anti-GFP (goat polyclonal)	Abcam	Ab6662	1:200
antibody	Anti-CTB (rabbit polyclonal)	Invitrogen	PA125635	1:200
antibody	Anti-mCherry (rabbit polyclonal)	Abcam	Ab183628	1:200
antibody	Anti-vGluT1 (mouse monoclonal)	Merck	MAB5502	1:200
antibody	Anti-NF (mouse monoclonal)	Abcam	Ab82259	1:500
antibody	Anti-syn (rabbit monoclonal)	Abcam	Ab32127	1:500
antibody	Anti-Akt (rabbit monoclonal)	Cell Signaling technology	4691s	1:200
antibody	Anti-p-Akt (rabbit	Cell Signaling technology	4060	1:200

	monoclonal)			
antibody	Anti-c-Fos (rabbit monoclonal)	Cell Signaling Technology	2250S	1:200
antibody	488-conjugated anti-goat (donkey polyclonal)	Abcam	Ab150129	1:800
antibody	488-conjugated anti-rabbit (goat polyclonal)	Abcam	Ab150077	1:800
antibody	647-conjugated anti-rabbit (donkey polyclonal)	Abcam	Ab150075	1:800
antibody	488-conjugated anti-mouse (goat polyclonal)	Abcam	Ab150117	1:800
antibody	555-conjugated anti-mouse (goat polyclonal)	Abcam	Ab150118	1:800
antibody	555-conjugated anti-rabbit (goat polyclonal)	Abcam	Ab150078	1:800
dyestuffs	α-BTX-555	Thermo Fisher Scientific	B35451	1:1000
recombinant DNA reagent	RV-N2C (G) -ΔG-EGFP	BrainVTA	R03001	
recombinant DNA reagent	AAV2/9-EF1α-DIO-oRVG-WPRE-hGH-pA	BrainVTA	PT-0023	

recombinant DNA reagent	AAV2/2Retro-EF1a-GCaMp6m-WPRE-hGH-pA	BrainVTA	PT-1700	
recombinant DNA reagent	rAAV-hSyn-GcaMP6f	Brain Case	BC-0079	
recombinant DNA reagent	AAV2/2Retro-nEf1α-mCherry-WPRE-pA	BrainVTA	PT-0099	
recombinant DNA reagent	AAV2/2Retro-hSyn-EGFP-WPRE-pA	Taitool Bioscience	S0237-2R	
recombinant DNA reagent	AAV2/9-hSyn-DIO-mCherry	Brain Case	BC-0025	
recombinant DNA reagent	CTB	Thermo Fisher Scientific	C34775	
recombinant DNA reagent	rAAV-hSyn-iGluSnFR3.v857.GPI	Brain Case	BC-0975	
recombinant DNA reagent	rAAV-hSyn-iGABASnFR	Brain Case	BC-0318	
recombinant DNA reagent	pAAV-CAG-EGFP-3xF-LAG-WPRE	Obio Technology	AOV002	
chemical compound, drug	TRDA	Thermo Fisher Scientific	D3328	
chemical compound, drug	AP5	Sigma	A8054	
chemical compound, drug	PTZ	Sigma	P6500	
chemical compound, drug	CNQX	MedChemExpress	HY-15066	
chemical	TTX	Sigma	554412	

compound, drug				
chemical compound, drug	Tizanidine	MedChemExpress	HY-B0194	
software, algorithm	GraphPad Prism 8.0	GraphPad Software		
software, algorithm	Origin 2018	OriginLab		
software, algorithm	Imaris 9.6	Oxford Instruments		
software, algorithm	ZEN 2012	ZEISS		
software, algorithm	MATLAB	Mathworks		

Supplementary table 2. Differential genes associated with Neuron axon development, Synaptic function, Inflammation and apoptosis

Dpysl2	Dll4	Spg11	Zfp36	Acvrl1	Gpr4
Bin3	Slc23a2	Ucp2	Frrs1l	Hk2	Dnm2
Dab2	Lef1	Ccar2	Tet1	Il4	Eng
Ripk3	Trim2	Grn	Gap43	Calm1	S100b
Matn2	Mcub	Sbno2	Cracr2b	Ramp2	Ctla2a
Myc	Lmna	Igfbp7	Fgfbp1	Ltbp1	Sema3g
Adcy8	Snx27	Gpi1	F2r	Hltf	Kdm4a
Enpp2	Bcl10	Calm2	Prex2	Cdkn1b	Atf3
Hes1	Ptprd	Plxnb2	Orai1	Ier3	Ugt8a
Arc	Spaar	Slitrk2	Lig4	Hmox1	Npy5r
Zbtb20	Ak4	Lrg1	Fzd4	Insr	Vcl
Slc11a2	Tnfrsf1b	Enpp1	Crh	Mmp8	Notch1
Cdkn1a	Mfsd2a	Icam1	Sarm1	Inpp5k	Cfh
Wfdc1	Bst1	Cib2	Rictor	Ggt1	Atp2c1
Slc25a27	Flt1	Lzts3	Map1b	Nfib	Gjc2
Epb41l3	Hsph1	Tgm2	Zfp212	Rala	Fam107a
Xdh	Spacdr	Ormdl3	Socs3	Nf2	Lcn2
Cacna1h	Ptn	Atg5	Gper1	Elk1	Hdac4
Adcyap1	Bhlhe40	Higd1a	Lifr	Trpv4	Ephb1
Dpysl3	Tnfrsf1a	Lpar1	Hmgb2	Gata2	Trpv3
Pmaip1	Pglyrp1	Trps1	S100a8	Serinc3	Tppp
Fbn2	Calca	Akna	S100a9	Myo1c	Hspa1a
Pdgfrb	Itgax	Micu3	Mpz	Ptgis	Fn1
Anxa1	Adm	Grin3a	C1qtnf3	Sgk1	Slc37a4
Fas	Abcc6	Cited2	Grin2a	Ccn2	Nrep
Il33	Stim1	Hip1	Hspa2	Unc5b	Bnip3
Plce1	Mcf2	Slc7a5	Cxcl12	Ddit4	Ctsl
Nptx1	Flna	Lrp1	Qki	Pfkl	Ctdsp1
Sox17	Polb	Mul1	Nrg1	Jade2	Cul5
Sgk3	Slit2	Shtn1	Lgi1	Pafah1b1	Id1
Nrp2	Slc7a2	Cldn5	Rap1a	Nfkbia	Fat3
Il1r1	Mt1	Atf4	Ahnak	Hsp90aa1	Mcur1
Sema4c	Jak3	Itpr3	Xlr3b	Edn1	

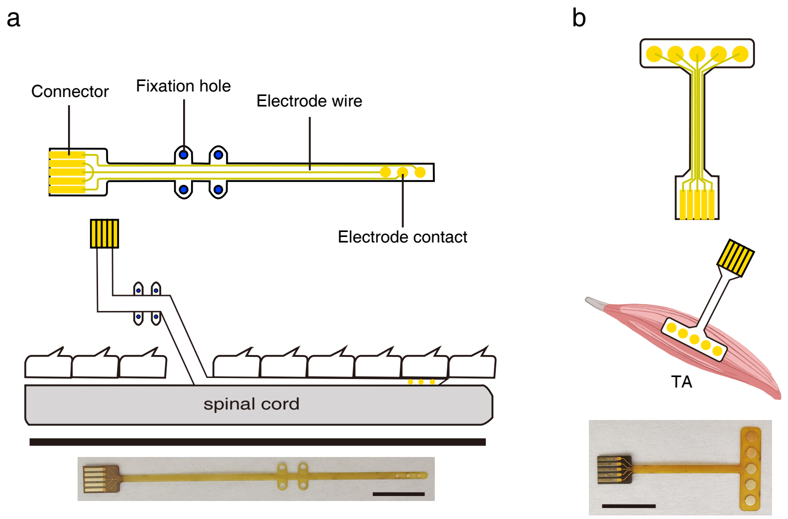

Fig. S1 | Epidural stimulation electrode and muscle stimulation electrode. a Schematic diagram of the epidural stimulation electrode. Yellow shading represents the interface connected to the stimulator. Solid yellow lines represent the electrode wires. Yellow circles denote the electrode contacts. Blue circles denote the electrode fixation holes. Scale bar, 5 mm. **b** Schematic diagram of the muscle stimulation electrode. Scale bar, 5 mm. Schematics were created with BioRender.com.

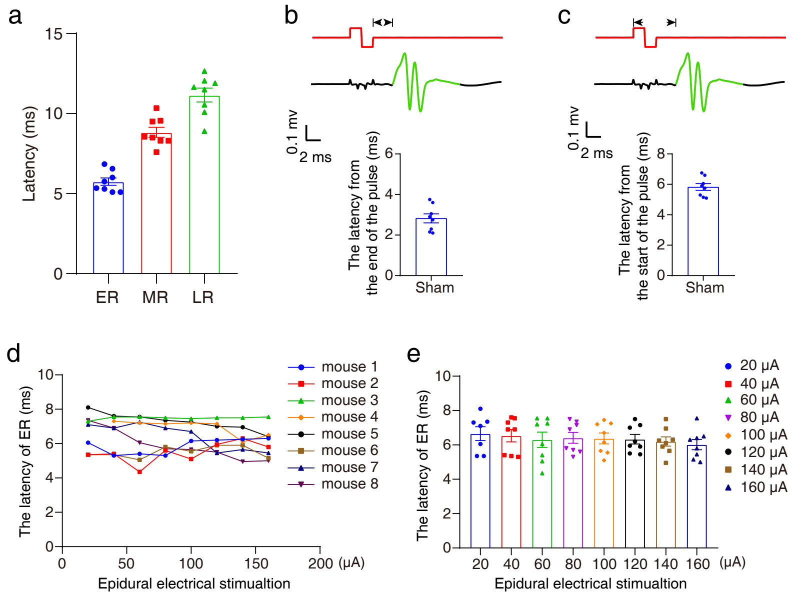

Fig. S2 | Parameters of the SCEP in sham mice. a The latency for ERs, MRs, and LRs of SCEPs in sham mice (n = 8 mice per group). **b** The latency from the end of the EES pulse to the SCEP (n = 8 mice). **c** The latency from the start of the EES pulse to the SCEP (n = 8 mice). **d** and **e** The latency for ERs in response to EES of different intensities (n = 8 mice per intensity).

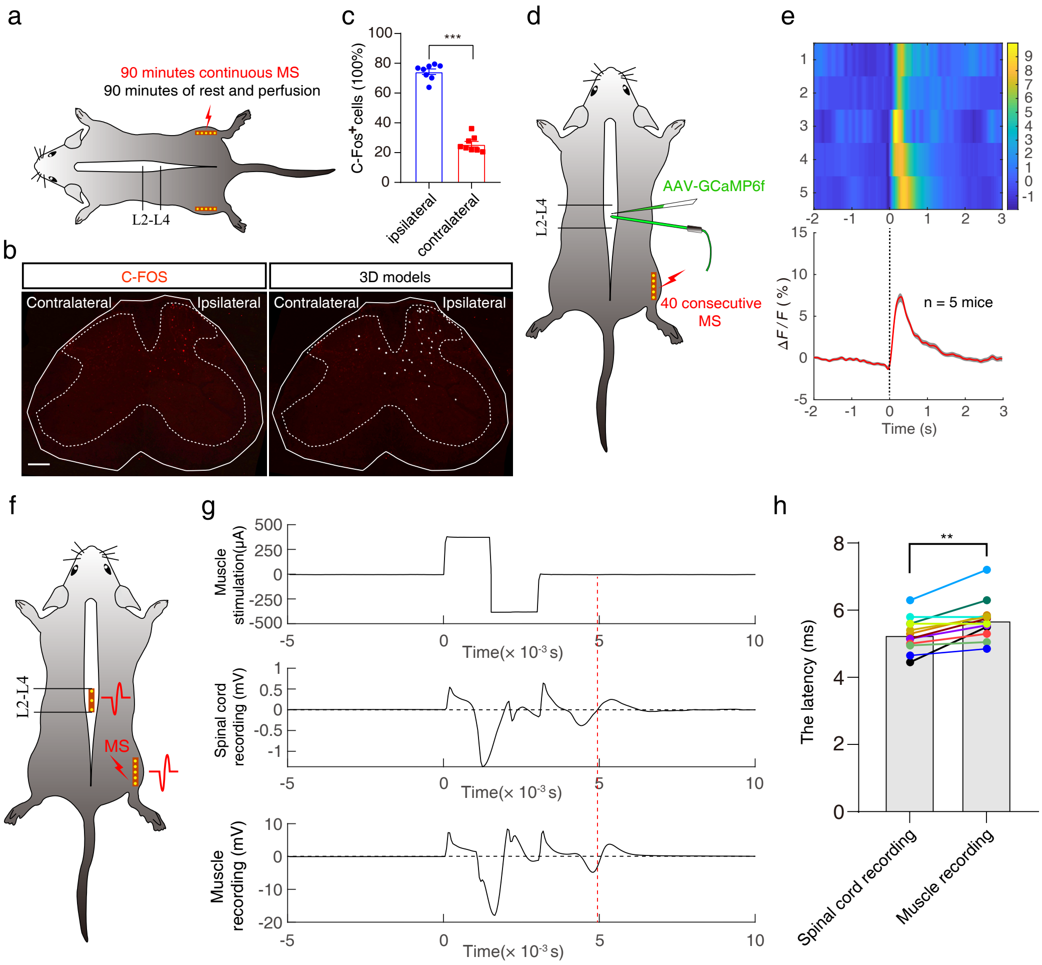

Fig. S3 | MS directly activates sensory fibers. a Experimental scheme. **b** Representative images showing the c-Fos immunostaining. Scale bar, 200 µm. **c** Ratio of c-Fos^{+} neurons in the ipsilateral spinal cord and contralateral spinal cord after MS (n = 8 mice per group). **d** Schematic of MS and Ca^{2+} fluorescence recording experiments. **e** Ca^{2+} signals associated with MS in mice. Upper, heatmap of Ca^{2+} signals aligned with the initiation of MS. Each row represents a mouse. Color scale at the right indicates *ΔF/F*. Lower, plot of the average Ca^{2+} transients. Thick lines indicate the mean, and shaded areas indicated the SEM. The vertical dotted line indicates the start of MS (n = 5 mice). **f** Experimental scheme. **g** During muscle stimulation, evoked signals were recorded in the spinal cord and muscles. **h** The latency of evoked signals recorded in the spinal cord and muscles after MS (n = 12 mice per group). Schematics in **a, d** and **f** were created with BioRender.com. Data presented in **c**, **e**, and **h** represent the mean ± SEM; statistical analysis was carried out with a two-tailed unpaired *t*-test (**c**) or a two-tailed paired *t*-test (**h**), $^{**}p < 0.01$, $^{***}p < 0.001$.

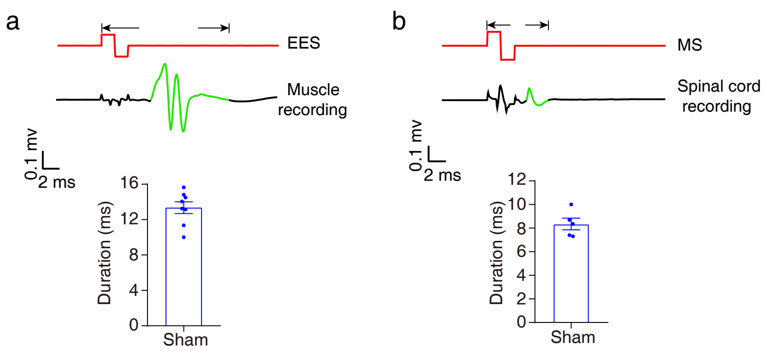

Fig. S4 | Transmission time of EES and MS signals in the sham mice. **a** Top: The red line shows EES; Middle: The waveform indicates the evoked signal recorded in the muscle. Bottom: The duration from the start of the spinal pulse to the end of the muscular response (n = 8 mice). **b** Top: The red line shows the MS; Middle: The waveform indicates the evoked signal recorded in the spinal cord. Bottom: The duration from the start of the muscular pulse to the end of the spinal response (n = 8 mice).

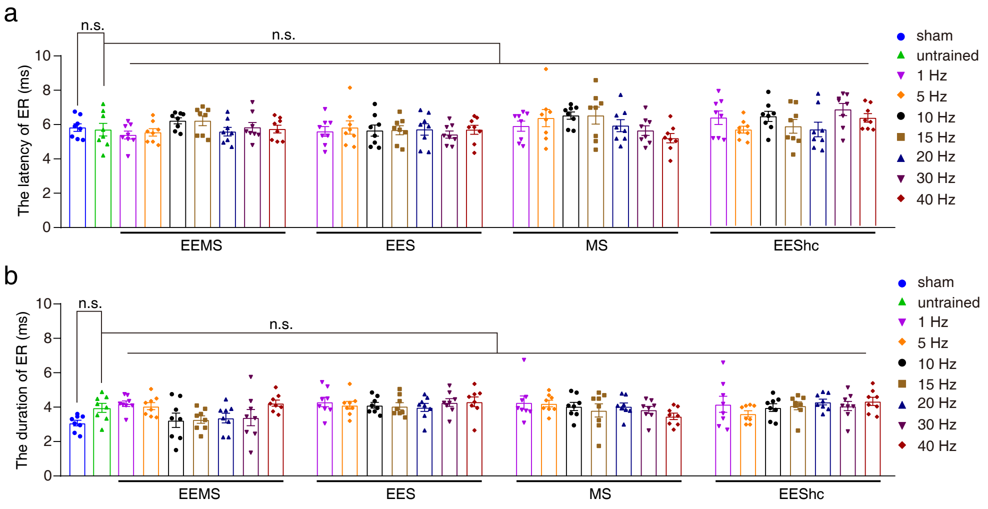

Fig. S5 | The latency and duration of ERs. a and **b** Histograms reporting the latency (**a**) and duration (**b**) of ERs in the sham, untrained, 1- to 40-Hz EEMS, 1- to 40-Hz EES, 1- to 40-Hz MS, and 1- to 40-Hz EEShc groups 29 days after electrode implantation (n = 8 mice per group). Data represent the mean ± SEM; ns: no statistically significant difference, one-way ANOVA followed by the Bonferroni post hoc test.

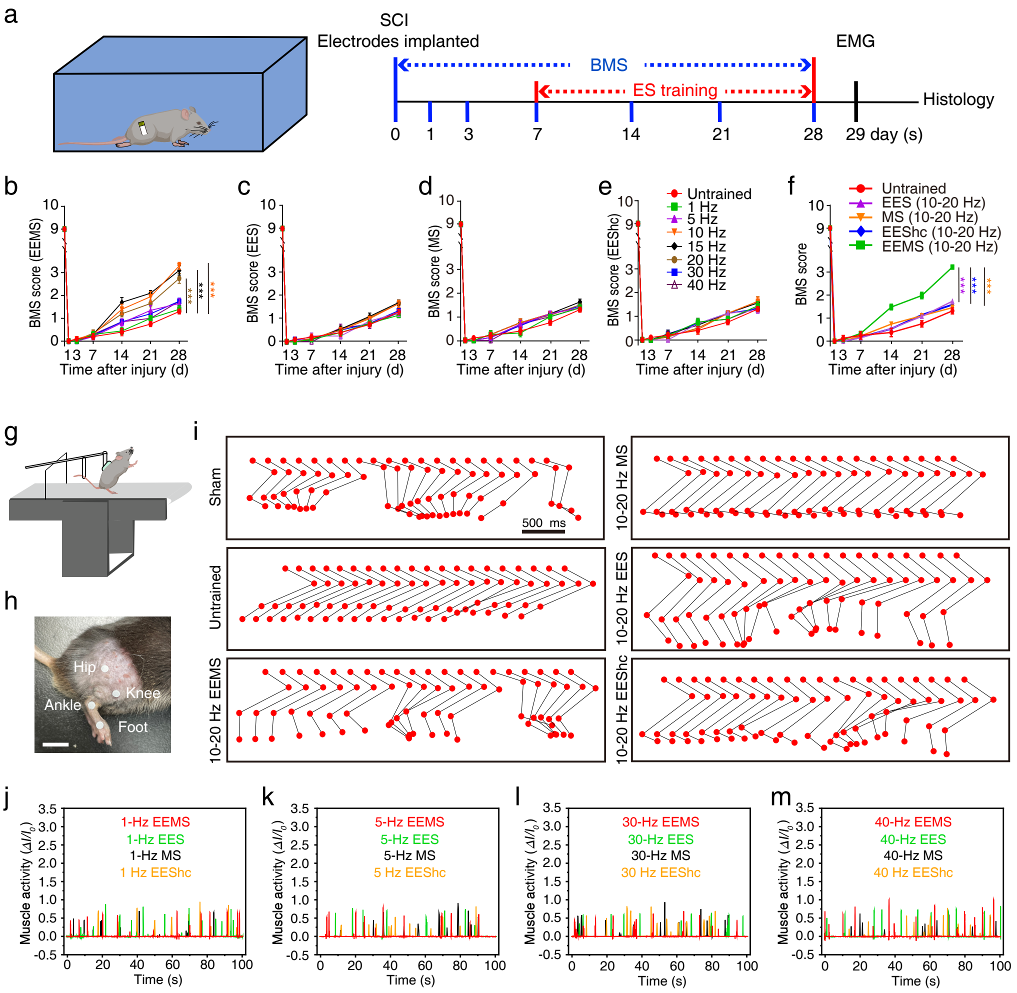

Fig. S6 | Overall motor function of the hindlimb in SCI mice. a Experimental scheme. **b-e** Hindlimb BMS scores of mice in the untrained, 1- to 40-Hz EEMS (**b**), 1- to 40-Hz EES (**c**), 1- to 40-Hz MS (**d**), and 1- to 40-Hz EEShc (**e**) groups at different time points after SCI (n = 8 mice per group). **f** Comparison of weighted averages of the hindlimb BMS scores of mice in the untrained, 10–20 Hz EES, 10–20 Hz MS, 10–20 Hz EEShc, and 10–20 Hz EEMS groups at different time points after SCI (n = 8 mice for the untrained group, n = 24 mice for the 10–20 Hz EES, 10–20 Hz MS, 10–20 Hz EEShc, and 10–20 Hz EEMS groups). **g** EMG of the TA muscle was carried out while mice walked on a treadmill. **h** Marking of the hip, knee joint, ankle joint, and sole of the foot of each mouse hindlimb. Scale bar, 1 cm. **i** Representative stick diagrams of hindlimb movements in the sham, untrained, 10-20 Hz EEMS, 10-20 Hz EES, and 10-20 Hz EEShc groups (n = 6 mice per group). **j-m** TA muscle contraction curve for mice in the EEMS (red), EES (green), MS (black), and EEShc (orange) groups (1 Hz, 5 Hz, 30 Hz, 40 Hz). Schematics in **a** and **g** were created with BioRender.com. Data represent the mean ± SEM; ns: no statistically significant difference, two-way ANOVA followed by the Tukey post hoc test (**b-f**), ***$p < 0.001$.

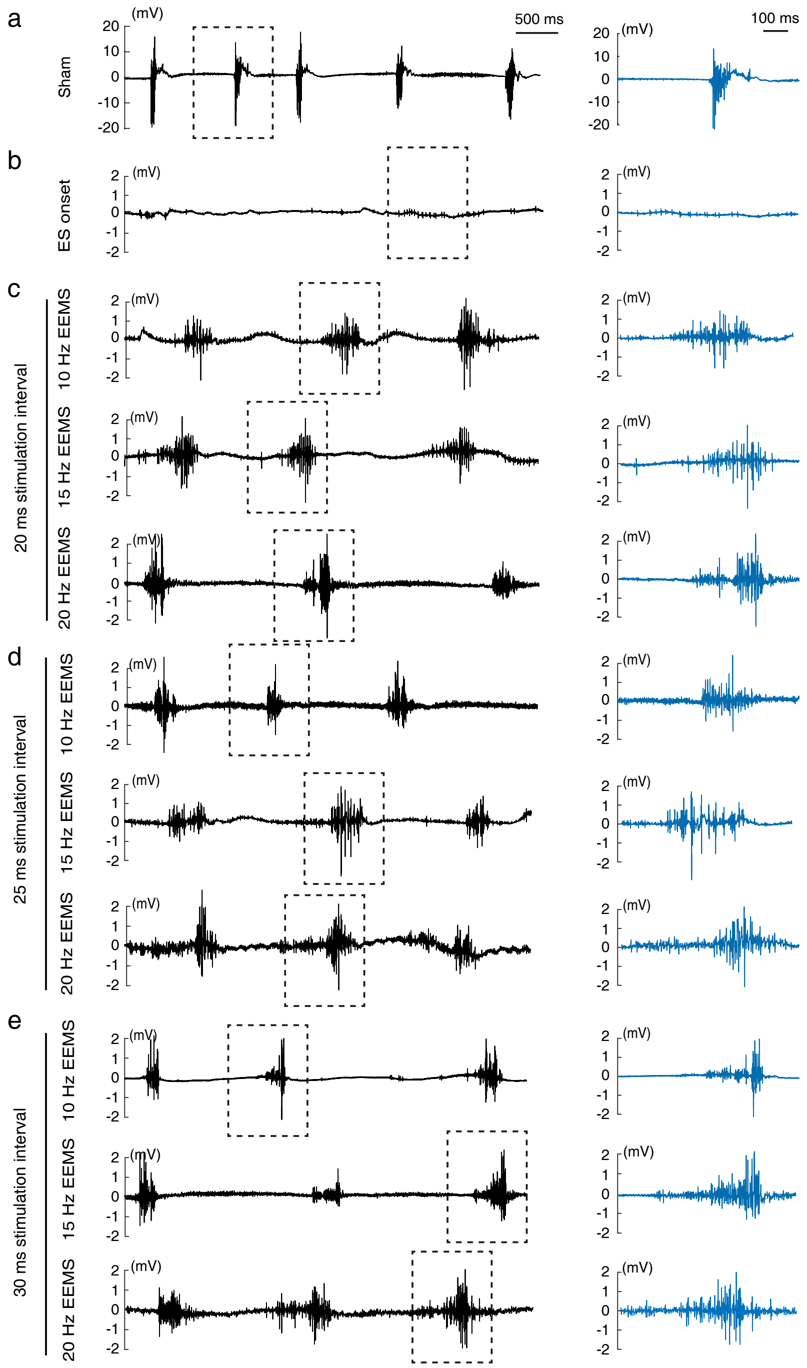

Fig. S7 | EMG after EEMS training with different stimulation intervals. a and **b** Left (black): Surface EMGs for TA muscles in the sham (**a**) and ES onset (**b**) groups over a period 5 s. Right (blue): enlarged view of the EMGs burst in the dashed box on the left. **c** Left (black): Surface EMGs of the TA muscles in the 10- to 20- Hz EEMS groups (20-ms interval between EES and MS) over a 5-s period. Right (blue), enlarged view of the EMGs burst in the dashed box on the left. **d** Left (black): Surface EMGs of the TA muscles in the 10- to 20- Hz EEMS groups (25-ms interval between EES and MS) over a 5-s period. Right (blue): enlarged view of the EMGs burst in the dashed box on the left. **e** Left (black): Surface EMGs of the TA muscles in the 10- to 20- Hz EEMS groups (30-ms interval between EES and MS) over a 5-s period. Right (blue):

enlarged view of the EMGs burst in the dashed box on the left. Mice from the sham group exhibited 5 steps within 5 s, whereas mice in the ES onset group exhibited 2 steps, and mice in the training group exhibited 3 steps (n = 6 mice per group).

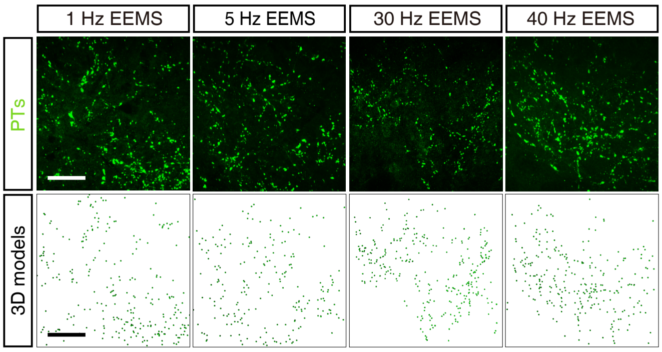

Fig. S8 | Projection of proprioceptive axons to the spinal cord in SCI mice after ineffective EEMS. CTB was injected into the TA muscle of SCI mice in the 1-Hz, 5-Hz, 30-Hz, and 40-Hz EEMS groups, the terminals of the proprioceptive axon were labeled (upper images), and 3D modeling was performed (lower images). Scale bar, 50 μm.

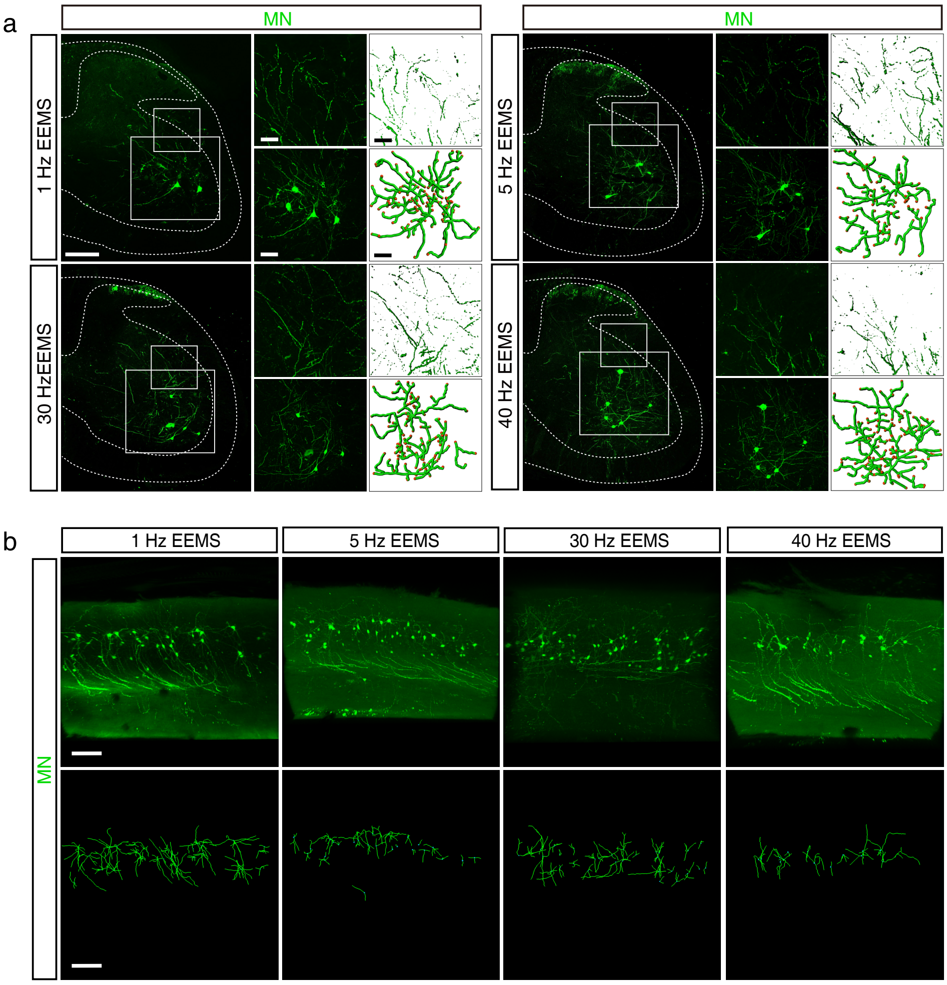

Fig. S9 | Morphological structure of motoneurons in SCI mice after ineffective EEMS. a Left, spinal motoneurons of SCI mice in the 1-Hz, 5-Hz, 30-Hz, and 40-Hz EEMS groups. Scale bar, 200 µm. Upper right, higher-magnification images and 3D models of the dendrites in the small boxed area. Scale bar, 50 µm. Lower right, partial images and 3D models of the motoneurons in the large boxed area. Scale bar, 100 µm. **b** Upper, spatial distribution of motoneurons of SCI mice in the 1-Hz, 5-Hz, 30-Hz, and 40-Hz EEMS groups. Scale bar, 200 µm. Lower, 3D models of the motoneurons. Scale bar, 200 µm.

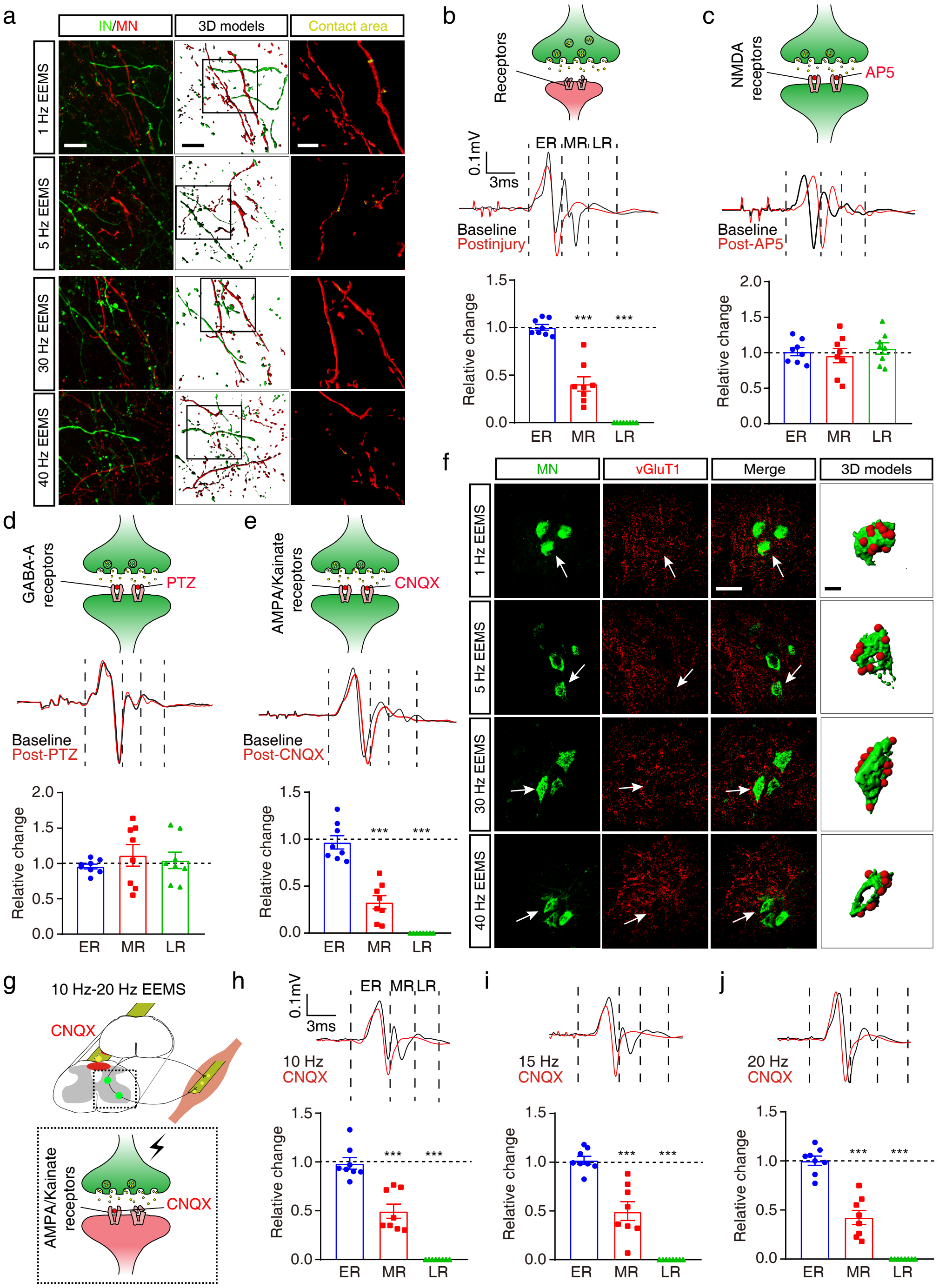

Fig. S10 | Remodeling of glutamate synaptic connections between spinal neurons in SCI mice after EEMS. a Left, synaptic connections between interneurons and motoneurons in the EEMS groups (1-Hz, 5-Hz, 30-Hz, 40-Hz). Scale bar, 20 μm. Middle, a 3D model of the picture on the left. Scale bar, 20 μm. Right, higher-magnification images of the boxed area. Yellow regions (overlap of GFP and mCherry) indicate regions of connection between interneurons and motoneurons. Scale bar, 10 μm. **b-e** Upper, diagram of synaptic connections between spinal neurons after SCI (**b**) and after administration of AP5 (**c**), PTZ (**d**), or CNQX (**e**) in intact mice; middle**,** SCEPs were recorded before and 1 week after SCI (**b**), and before and 30 min after intrathecal injection of AP5 (**c**), PTZ (**d**), or CNQX (**e**) in intact mice. Each waveform

was the average of 50 SCEP; lower, relative changes in ER, MR, and LR amplitude after SCI compared with the baseline (**b**), and compared with the baseline, the relative change of ER, MR, and LR amplitudes after intrathecal injection of AP5 (**c**), PTZ (**d**), or CNQX (**e**) (n = 8 mice per group). **f** Motoneurons were marked with CTB (green) and vGluT1 (red) to visualize axonal terminals of glutamatergic neurons of SCI mice in the 1-Hz, 5-Hz, 30-Hz, and 40-Hz EEMS groups. Scale bar, 50 µm. Bottom panels, 3D models of the motoneurons indicated by the white arrows and with vGluT1 on their surface. Scale bar, 10 µm. **g** Detecting SCEPs of SCI mice in the 10- to 20-Hz EEMS groups after CNQX administration. The dashed box denotes the synaptic connection between neurons after administration of CNQX. **h**-**j** SCEPs were recorded (upper traces) in the 10-Hz (**h**), 15-Hz (**i**), and 20-Hz (**j**) EEMS groups before and after administration of CNQX. Bar plots (lower graphs) shown the relative changes in ER, MR, and LR amplitudes before and after CNQX administration (n = 8 mice per group). Schematics in **b, c, d, e,** and **g** were created with BioRender.com. Data represent the mean ± SEM; ns: no statistically significant difference, ***p < 0.001, two-tailed unpaired t-test (**b**-**e** and **h**-**j**).

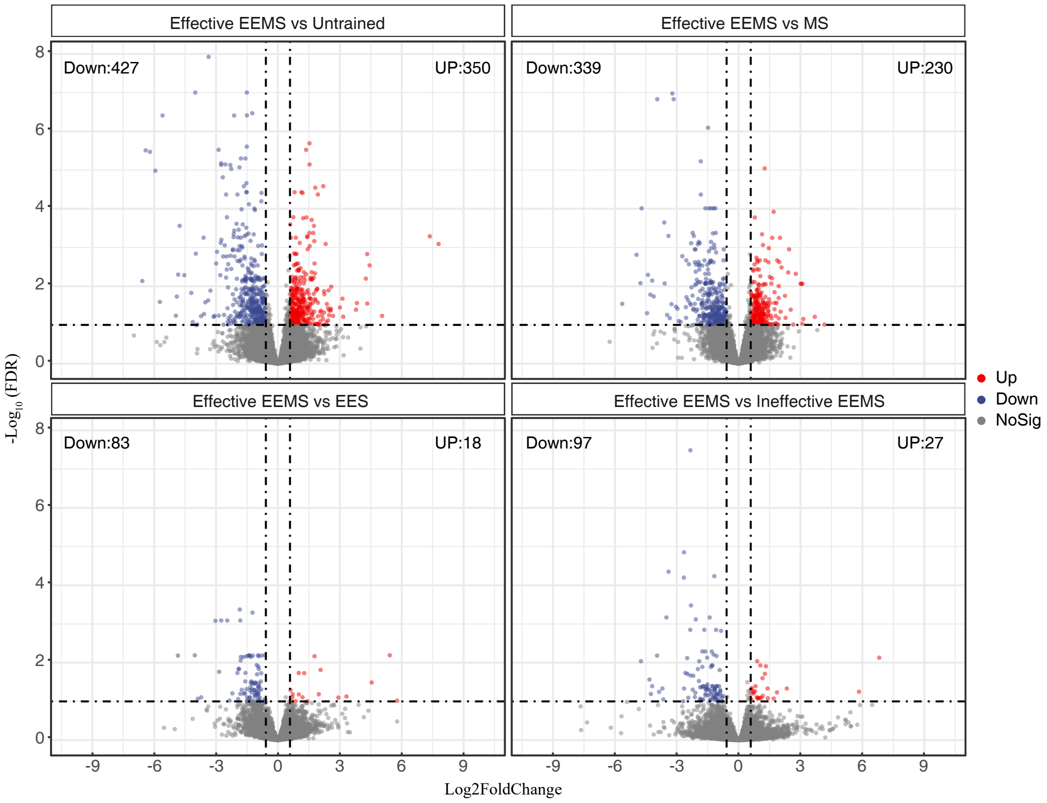

Fig. S11 | All differentially expressed genes associated with isolated spinal motoneurons. All differentially expressed genes (effective EEMS vs untrained, effective EEMS vs EES, effective EEMS vs MS, and effective EEMS vs ineffective EEMS). Red dots indicate upregulated genes (Up), gray dots indicate genes for which expression did not differ significantly, and blue dots indicate downregulated genes (Down).

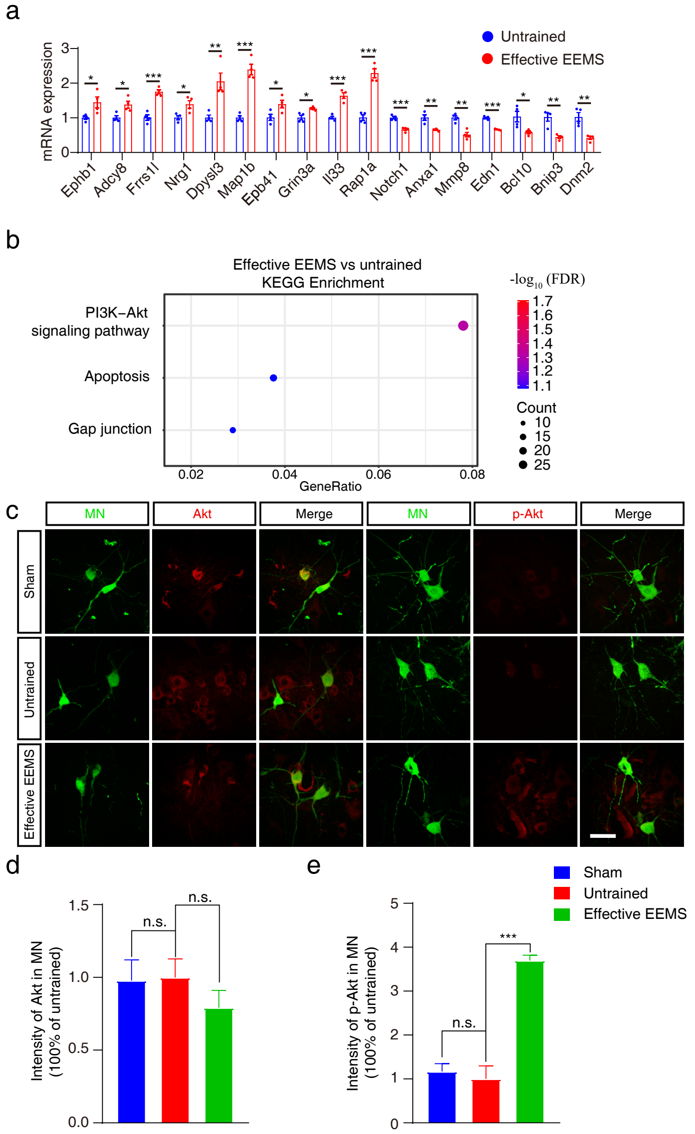

Fig. S12 | Validation of single cell transcriptome sequencing results. a Expression of mRNAs encoded by genes in **Fig. 9b** was verified by qPCR in the lumbar spinal cord (n = 4 mice per group). **b** Statistical analyses of signaling pathways from KEGG (Kyoto Encyclopedia of Genes and Genomes), featuring the functional distribution of related genes and the related signaling pathways that reached statistical significance between the effective EEMS group and untrained group. Dot color indicates the FDR ($-\log_{10}$ (FDR)), which ranged from 1.1 (blue) to 1.7 (red). **c** Coimmunostaining of Akt, p-Akt, and motoneurons in coronal sections in the sham, untrained, and effective EEMS groups. Scale bar, 50 µm. **d** and **e** Quantification of the expression of Akt (**d**), p-Akt (**e**) proteins in motoneurons (n = 5 mice per group). Data represent the mean ± SEM, ns: no statistically significant difference, **p* < 0.05, ***p* < 0.01, ****p* < 0.001, two-tailed unpaired *t*-test (**a**), one-way ANOVA followed by the Bonferroni post hoc test (**d** and **e**).

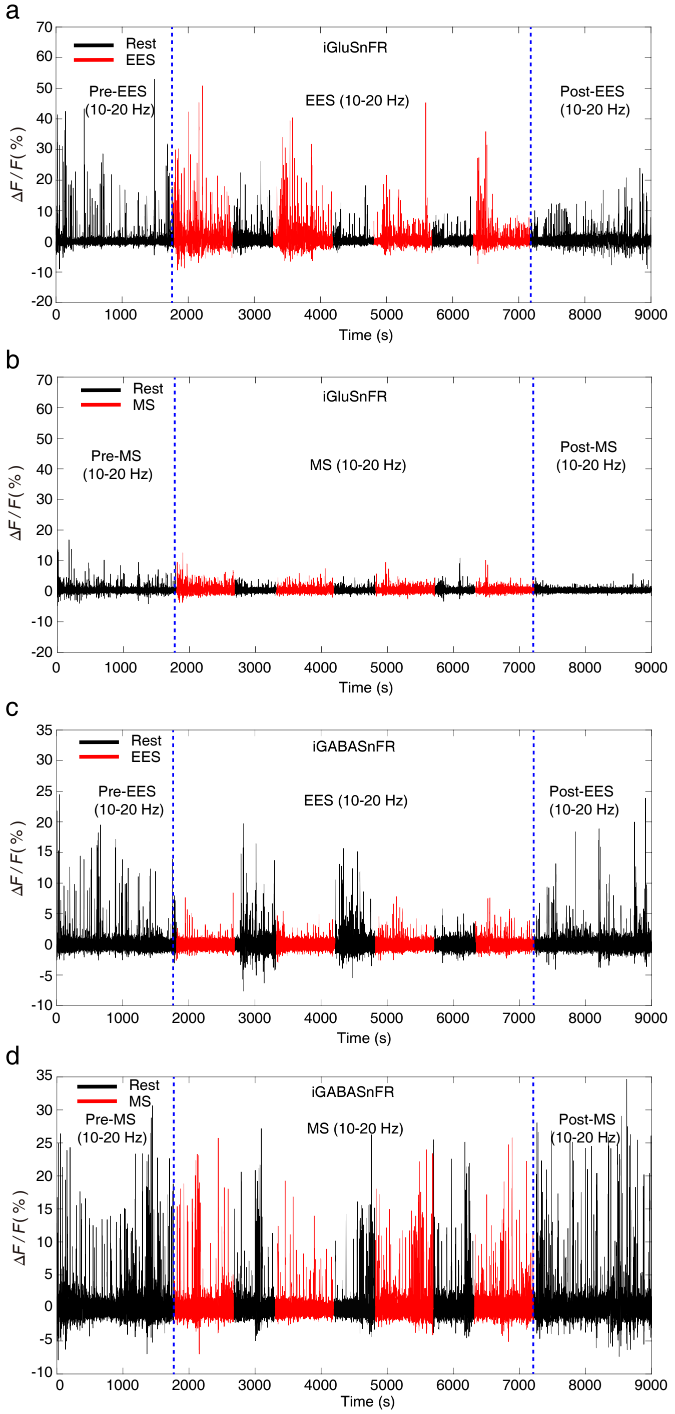

Fig. S13 | Effect of EES (10-20 Hz) and MS (10-20 Hz) on the neurotransmitters flow of Glu and GABA. a and **b** Burst curve of Glu before, during and post EES (10-20 Hz) (**a**) and MS (10-20 Hz) (**b**). **c** and **d** Burst curve of GABA before, during and post EES (10-20 Hz) (**c**) and MS (10-20 Hz) (**d**).
