# Supplementary material for: Dual electrical stimulation at spinal-muscular interface reconstructs spinal sensorimotor circuits after spinal cord injury
Source: Nat Commun. 2024 Jan 19;15:619. doi: 10.1038/s41467-024-44898-9 (PMC10799086; doi:10.1038/s41467-024-44898-9)
Supplement: Supplementary file 3 — Description of Additional Supplementary Files [file 41467_2024_44898_MOESM3_ESM.pdf]

Description of additional supplementary files

Supplementary Data 1: Primers used in this study (synthesized by Sangon Biotech)

Supplementary Movie 1: The movements of the hindlimbs of mice in three training groups and untrained group. The movie shows the hind limb movements of mice in the untrained and 20-40 Hz EES, 20-40 Hz MS, and 10-20 Hz EEMS groups at 3 and 18 days of electrical stimulation training.
