# Supplementary material for: Dual electrical stimulation at spinal-muscular interface reconstructs spinal sensorimotor circuits after spinal cord injury
Source: Nat Commun. 2024 Jan 19;15:619. doi: 10.1038/s41467-024-44898-9 (PMC10799086; doi:10.1038/s41467-024-44898-9)
Supplement: Supplementary file 6 — Reporting Summary [file 41467_2024_44898_MOESM6_ESM.pdf]

Corresponding author(s): Yaobo Liu

Last updated by author(s): Dec 9, 2023

Reporting Summary

Nature Portfolio wishes to improve the reproducibility of the work that we publish. This form provides structure for consistency and transparency in reporting. For further information on Nature Portfolio policies, see our Editorial Policies and the Editorial Policy Checklist.

Please do not complete any field with "not applicable" or n/a. Refer to the help text for what text to use if an item is not relevant to your study.
For final submission: please carefully check your responses for accuracy; you will not be able to make changes later.

Statistics

For all statistical analyses, confirm that the following items are present in the figure legend, table legend, main text, or Methods section.

n/a	Confirmed	
☐	☒	The exact sample size (*n*) for each experimental group/condition, given as a discrete number and unit of measurement
☐	☒	A statement on whether measurements were taken from distinct samples or whether the same sample was measured repeatedly
☐	☒	The statistical test(s) used AND whether they are one- or two-sided<br>*Only common tests should be described solely by name; describe more complex techniques in the Methods section.*
☒	☐	A description of all covariates tested
☐	☒	A description of any assumptions or corrections, such as tests of normality and adjustment for multiple comparisons
☐	☒	A full description of the statistical parameters including central tendency (e.g. means) or other basic estimates (e.g. regression coefficient) AND variation (e.g. standard deviation) or associated estimates of uncertainty (e.g. confidence intervals)
☐	☒	For null hypothesis testing, the test statistic (e.g. *F*, *t*, *r*) with confidence intervals, effect sizes, degrees of freedom and *P* value noted<br>*Give P values as exact values whenever suitable.*
☒	☐	For Bayesian analysis, information on the choice of priors and Markov chain Monte Carlo settings
☒	☐	For hierarchical and complex designs, identification of the appropriate level for tests and full reporting of outcomes
☒	☐	Estimates of effect sizes (e.g. Cohen's *d*, Pearson's *r*), indicating how they were calculated

Our web collection on statistics for biologists contains articles on many of the points above.

Software and code

Policy information about availability of computer code

Data collection	16-channel physiological signal recording and analysis system (MP150, BIOPAC, USA)<br>Confocal laser scanning microscopy (LSM700, Zeiss, Germany)<br>Neural signal recording system (QAXK-FPS, Thinkertech, China)<br>HD camera (FDR-AX30, Sony, Japan)<br>Oscilloscope (MSO72504DX, Tektronix, USA)<br>11.7 T MRI (Biospec 11.7 /16, Brugg, Germany)
Data analysis	For statistics, data were analysed using Graph Prism 8 (8.0.1,GraphPad Software, Inc., USA), Origin 2018 (2018C, OriginLab, USA). For microscopy, images were analysed using Imaris (9.6, Oxford instruments, England), Fiji(Windows 64,v1.51,NIH). Spinal Cord Evoked Potential (SCEP) data analysis was using MATLAB (R2018a, MathWorks, USA). Electromyography (EMG) data analysis was using 16-channel physiological signal recording and analysis system (MP150, BIOPAC, USA).

For manuscripts utilizing custom algorithms or software that are central to the research but not yet described in published literature, software must be made available to editors and reviewers. We strongly encourage code deposition in a community repository (e.g. GitHub). See the Nature Portfolio guidelines for submitting code & software for further information.

Data

Policy information about availability of data

All manuscripts must include a data availability statement. This statement should provide the following information, where applicable:

- Accession codes, unique identifiers, or web links for publicly available datasets
- A description of any restrictions on data availability
- For clinical datasets or third party data, please ensure that the statement adheres to our policy

The data to support the findings of this study are included in the paper and supplementary information. Source data are provided with this paper. Any additional requests for information can be directed to, and will be fulfilled by, the corresponding authors. The single cell transcriptome sequencing data generated in this study have been deposited in the GEO database under accession code at https://www.ncbi.nlm.nih.gov/geo/query/acc.cgi?acc=GSE243038.

Research involving human participants, their data, or biological material

Policy information about studies with human participants or human data. See also policy information about sex, gender (identity/presentation), and sexual orientation and race, ethnicity and racism.

Reporting on sex and gender	N/A
Reporting on race, ethnicity, or other socially relevant groupings	N/A
Population characteristics	N/A
Recruitment	N/A
Ethics oversight	N/A

Note that full information on the approval of the study protocol must also be provided in the manuscript.

Field-specific reporting

Please select the one below that is the best fit for your research. If you are not sure, read the appropriate sections before making your selection.

☒ Life sciences ☐ Behavioural & social sciences ☐ Ecological, evolutionary & environmental sciences

Life sciences study design

All studies must disclose on these points even when the disclosure is negative.

Sample size	Sample sizes were indicated in the legend of each Figure and Supplementary Figure. No statistical methods were used to predetermine sample size. Estimates were made based on references, experimental approach, availability and feasibility required to obtain statistically significant results.
Data exclusions	animals were excluded for the mice after surgery/died before the end of the study.
Replication	The experimental findings were reliably reproduced, for representative data used for statistical analysis, the number of animals or experiments is described in corresponding figure legends.
Randomization	As reported in Methods and figure legends, mice were randomly assigned to each group to receive training.
Blinding	For in vivo studies, experimenters were blinded to group assignment. Blinding was performed for immunohistochemistry, electrophysiological recording and analysis, and calcium signal quantification. Behavioural tests (BMS scores, motor trajectory testing, muscle strength testing) were determined from independent persons who were unaware of the expected therapeutic outcome.

Reporting for specific materials, systems and methods

We require information from authors about some types of materials, experimental systems and methods used in many studies. Here, indicate whether each material, system or method listed is relevant to your study. If you are not sure if a list item applies to your research, read the appropriate section before selecting a response.

Materials & experimental systems

n/a	Involved in the study	
☐	☒	Antibodies
☒	☐	Eukaryotic cell lines
☒	☐	Palaeontology and archaeology
☐	☒	Animals and other organisms
☒	☐	Clinical data
☒	☐	Dual use research of concern
☒	☐	Plants

Methods

n/a	Involved in the study	
☒	☐	ChIP-seq
☒	☐	Flow cytometry
☐	☒	MRI-based neuroimaging

Antibodies

Antibodies used

Primary antibodies against the following proteins were used as follows:
goat anti-GFP (Abcam, Ab6662, USA, 1:200)
rabbit anti-CTB (Invitrogen, PA125635, USA, 1:200)
rabbit anti-mCherry (Abcam, Ab183628, USA, 1:200)
mouse anti-vGluT1 (Merk Millipore, MAB5502, USA, 1:200)
mouse anti-NF(Abcam, Ab82259, USA, 1:500)
rabbit anti-syn(Abcam, Ab32127, USA, 1:500)
rabbit anti-akt(Cell Signaling technology, 4691s,USA, 1:200)
rabbit anti-p-akt(Cell Signaling technology, 4060,USA, 1:200)
rabbit anti-c-Fos (Cell Signaling technology, 2250S, 1:200)
Secondary antibodies:
488-conjugated anti-goat (Abcam, ab150129, 1:800)
488-conjugated anti-rabbit (Abcam, ab150077, 1:800)
647-conjugated anti-rabbit (Abcam, ab150075, 1:800)
488-conjugated anti-mouse (Abcam, ab150117, 1:800)
555-conjugated anti-mouse (Abcam, ab150118, 1:800)
555-conjugated anti-rabbit (Abcam, ab150078, 1:800)

Validation

Anti-GFP (Abcam, Ab6662, USA, 1:200), species: goat, reacts with: Species independent, suitable for: IHC-FoFr, IHC-Fr, WB, ICC/IF. Meijer M et al. Epigenomic priming of immune genes implicates oligodendroglia in multiple sclerosis susceptibility. Neuron 110:1193-1210.e13 (2022).
Anti-CTB (Invitrogen, PA125635, USA, 1:200), species: rabbit, reacts with: Bacteria, suitable for: ELISA.
Anti-mCherry (Abcam, Ab183628, USA, 1:200), species: rabbit, reacts with: Species independent, suitable for: ICC/IF, IHC - Wholemount, IP, WB. Zhang X et al. Exostosin glycosyltransferase 1 reduces porcine reproductive and respiratory syndrome virus infection through proteasomal degradation of nsp3 and nsp5. J Biol Chem 298:101548 (2022).
Anti-vGluT1 (Merk Millipore, MAB5502, USA, 1:200), species: mouse, reacts with: Rat, suitable for: IHC, WB. Fazzari P et al. Cell autonomous regulation of hippocampal circuitry via Aph1b-γ-secretase/neuregulin 1 signalling. Elife. 2014 Jun 2;3:e02196.
Anti-NF(Abcam, Ab82259, USA, 1:500), species: mouse, reacts with: Mouse, Rat, Human, suitable for: WB, IHC-P, IHC-Fr, IHC-FoFr Flow Cyt, ICC/IF.
Anti-syn(Abcam, Ab32127, USA, 1:500), species: rabbit, reacts with: Mouse, Rat, Human, suitable for: WB, IHC-P, ICC/IF. Li ZD et al. The divergent effects of astrocyte ceruloplasmin on learning and memory function in young and old mice. Cell Death Dis 13:1006 (2022).
Anti-akt(Cell Signaling technology, 4691s,USA, 1:200), species: rabbit, reacts with: Human, Mouse, Rat, Monkey, D. melanogaster, suitable for: WB, IHC-P, ICC/IF, IP, FC. Bi Y et al. A novel FGFR1 inhibitor CYY292 suppresses tumor progression, invasion, and metastasis of glioblastoma by inhibiting the Akt/GSK3β/snail signaling axis. Genes Dis. 2023 Apr 3;11(1):479-494.
Anti-p-akt(Cell Signaling technology, 4060,USA, 1:200), species: rabbit, reacts with: Human, Mouse, Rat, Monkey, D. melanogaster, Hamster, Zebrafish, Bovine, suitable for: WB, SW, IHC-P, ICC/IF, IP, FC. Voelkl K et al. Neuroprotective effects of hepatoma-derived growth factor in models of Huntington's disease. Life Sci Alliance. 2023 Aug 14;6(11):e202302018.
Anti-c-Fos (Cell Signaling technology, 2250S, 1:200), species: rabbit, reacts with: Human, Mouse, Rat, suitable for: WB, SW, IFF, IF, CIP, FC. Yang Y et al. Microglia are involved in regulating histamine-dependent and non-dependent itch transmissions with distinguished signal pathways. Glia. 2023 Nov;71(11):2541-2558.
488-conjugated anti-goat (Abcam, ab150129, 1:800), species: goat, Host: Donkey, suitable for: IHC-Fr, ICC/IF, Flow Cyt, IHC-P, ELISA. Widjaja AA et al. Targeting endogenous kidney regeneration using anti-IL11 therapy in acute and chronic models of kidney disease. Nat Commun 13:7497 (2022).
488-conjugated anti-rabbit (Abcam, ab150077, 1:800), species: rabbit, Host: Goat, suitable for: ICC/IF, Flow Cyt, IHC-P, ELISA, IHC-Fr. Li X et al. Electrical charge on ferroelectric nanocomposite membranes enhances SHED neural differentiation. Bioact Mater 20:81-92 (2023).
647-conjugated anti-rabbit (Abcam, ab150075, 1:800), species: rabbit, Host: Donkey, suitable for: ICC/IF, ELISA, IHC-P, Flow Cyt, IHC-Fr. Ji Y et al. N6-Methyladenosine Modification of CIRCKRT17 Initiated by METTL3 Promotes Osimertinib Resistance of Lung Adenocarcinoma by EIF4A3 to Enhance YAP1 Stability. Cancers (Basel) 14:N/A (2022).
488-conjugated anti-mouse (Abcam, ab150117, 1:800), species: mouse, Host: Goat, suitable for: IHC-Fr, ICC/IF, Flow Cyt, IHC-P, ELISA. Weichselberger V et al. Eya-controlled affinity between cell lineages drives tissue self-organization during Drosophila oogenesis. Nat Commun 13:6377 (2022).
555-conjugated anti-mouse (Abcam, ab150118, 1:800), species: mouse, Host: Goat, suitable for: IHC-Fr, ICC/IF, ELISA, IHC-P, Flow Cyt. Chen Y et al. Fibroblast growth factor 20 attenuates pathological cardiac hypertrophy by activating the SIRT1 signaling pathway. Cell Death Dis 13:276 (2022).
555-conjugated anti-rabbit (Abcam, ab150078, 1:800), species: rabbit, Host: Goat, suitable for: IHC-Fr, ICC/IF, ELISA, IHC-P, Flow Cyt.

Bony AR et al. Analgesic α-Conotoxin Binding Site on the Human GABAB Receptor. Mol Pharmacol 102:196-208 (2022).

Animals and other research organisms

Policy information about studies involving animals; ARRIVE guidelines recommended for reporting animal research, and Sex and Gender in Research

Laboratory animals	C57B6L/J mive (about 8 weeks, 25-30 g) were purchased from the Shanghai SLAC Laboratory Animal Company (Shanghai, China). ChAT-cre mice (about 8 weeks, 25-30 g) were kindly provided by Dr. Zilong Qiu (Institute of Neuroscience, Chinese Academy of Sciences, Shanghai, China). Lbx1-cre mice (about 8 weeks, 25-30 g) were constructed by GemPharmatech and maintained on a mixed genetic background (129/C57Bl6). All animals were housed in a specific pathogen-free environment at ambient temperature (24 ± 2℃), air humidity 40- 70% and 12 h dark/12 h light cycle.
Wild animals	No wild animals were used in the study.
Reporting on sex	According to previous experimental method, male mice were used in all experiments.
Field-collected samples	This study did not involve any sample collected from the field.
Ethics oversight	Animal Ethics Committe of Soochow University

Note that full information on the approval of the study protocol must also be provided in the manuscript.

Magnetic resonance imaging

Experimental design

Design type	Structural imaging of the spinal cord in a single mouse.
Design specifications	No functional MRI was performed. Four weeks after the spinal cords of mice were implanted with electrodes, structural MRI imaging of the thoracic and lumbar segments of the spinal cords of mice was performed in order to detect the position of the electrodes in the spinal cord. The scanned images were not post-processed. One mouse in good condition was examined.
Behavioral performance measures	No behavioral measures were performed during MRI scans as the animals were anesthetized.

Acquisition

Imaging type(s)	Structural
Field strength	11.7 Tesla
Sequence & imaging parameters	The imaging parameters were as follows: repetition time (TR) =2000 ms, echo time (TE) =26 ms, field of view (FOV) =3 cm × 3 cm, matrix size =128 × 128, slice thickness = 0.5 mm, and cumulative scans performed four times. To minimize the influence of respiratory motion on the results, a respiratory gating technique was employed.
Area of acquisition	The imaging range included the thoracic and lumbar segments of the spinal cord.

Diffusion MRI ☐ Used ☒ Not used

Preprocessing

Preprocessing software	The data were not processed and were obtained directly from MRI scans.
Normalization	Single images were obtained directly from MRI scans. It was not normalised.
Normalization template	no standardized template was used.
Noise and artifact removal	Single images were obtained directly from MRI scans to visualise the spinal cord and electrode positions. It has not been post-processed.
Volume censoring	No volume censoring was employed

Statistical modeling & inference

Model type and settings	The original images were obtained directly from the MRI scans and were not processed in any way.
Effect(s) tested	No tasks or stimuli tested. ANOVA was not used.

Specify type of analysis: ☐ Whole brain ☒ ROI-based ☐ Both

Anatomical location(s): spinal cord

Statistic type for inference<br>(See Eklund et al. 2016)	Single images were acquired directly from the MRI scan to show the spinal cord and electrode locations. Images were not statistically analysed.
Correction	Single images were acquired directly from the MRI scan to show the spinal cord and electrode locations. Images were not statistically analysed.

Models & analysis

n/a	Involved in the study
☒	☐ Functional and/or effective connectivity
☒	☐ Graph analysis
☒	☐ Multivariate modeling or predictive analysis

This checklist template is licensed under a Creative Commons Attribution 4.0 International License, which permits use, sharing, adaptation, distribution and reproduction in any medium or format, as long as you give appropriate credit to the original author(s) and the source, provide a link to the Creative Commons license, and indicate if changes were made. The images or other third party material in this article are included in the article's Creative Commons license, unless indicated otherwise in a credit line to the material. If material is not included in the article's Creative Commons license and your intended use is not permitted by statutory regulation or exceeds the permitted use, you will need to obtain permission directly from the copyright holder. To view a copy of this license, visit http://creativecommons.org/licenses/by/4.0/
